# Necrosis

**Published:** 1866-01

**Authors:** J. W. P. Bates


Reported J. W. P. Bates, M. D.

### NECROSIS.

Boy, æt. 17. The term necrosis implies mortification of bone. Bone mortifies like soft parts; perishes from diseased periosteum; often dies, as a result of scrofulous inflammation. Bone is vascular, and invested by a vascular fibrous membrane, called the periosteum, which bears the same relation to bone that bark does to a tree; when a bone is fractured, this membrane is torn. It is also lined by an areolar vascular membrane, which is sometimes called the internal periosteum. This internal membrane is quite sensitive; the medulla itself is not sensitive. The bones are vascular, but depend upon the blood vessels they receive from the external or internal periosteum. When, by progress of disease, a portion of these membranes is stripped off, the bone perishes, and we have necrosis. We have a familiar example of this in the common felon—matter forms beneath the theca of the tendons, and the bone dies. The long bones are most generally affected, When matter forms between external membrane and the bone, it is very likely to occur on internal membrane also. When the matter is discharged, the patient gets immediate relief, but the bone is dead and is called sequestrum. Its extremities are very irregular ; and it is an inevitable result that dead bone will separate itself from the living. It may maintain its form and office, but in course of time it must become detached. For a time, continuity is complete, but after a while it separates, and if no provision were made for such a contingency by nature, we would have spontaneous fracture, and in some rare instances this does occur. The process of repair in these cases is analogous to the repair of fractures. The detached periosteum pours out callus, which surrounds the dead bone. Always suppuration wherever dead bone is present, and there will be fenestra for the discharge of the matter, and through these we can touch the sequestrum, and may introduce strong forceps and extract it. Sometimes it may come out by the efforts of nature alone, but in a great many instances it is necessary to cut down on the callus and chisel away the new bone, and extract the sequestrum, or break it. The cavity will fill up and the external callus will be absorbed to a considerable extent, but there almost always remains some enlargement. Necrosis rarely extends into a joint, because the head and shaft of a bone derive their vascular supply from different points ; it is also dependent upon the different points of ossification. When it occurs in cubical bone, as in this case, there is not that vital distinction between extremities and shaft, and is more disastrous in its effects, more likely to penetrate joints, and not so well circumscribed.

Here the astragalus is diseased. On introducing a probe, I think I feel dead bone. We will cut down upon it, and even if it is not in such a condition that we can extract it readily, the incision will give relief by relieving tension, and the loss of blood will deplete the parts of some of their superabundant blood. I do not expect to find a great amount of loose bone. A small piece was extracted. We will have this wound packed with lint, to keep it expanded. Carious bone may be all that is remaining—this corresponds to ulceration of the soft parts—and to counteract this, we will inject with a solution of muriatic acid, from time to time, which will dissolve the earthy matter, and make a salt soluble in the discharges, and easily got rid of in this way.

Nov. 8. Case doing very well—not so much pain as he had. Seems somewhat weak, so we will order

R. Syr. ferri. iod., gtt. xx., ter die.

---

*Punch* has recently informed his readers that *Io dide* of Potassium. *Vanity Fair* told the American world the same thing four years ago; and "Meister Karl" claims that he was the first who discovered a chemical formula in the Bible, where water is correctly described as consisting of Hydrogen and Oxygen in the text, "HO every one that thirsteth!"

